# The legitimacy of pain according to sufferers

**DOI:** 10.1371/journal.pone.0291112

**Published:** 2023-11-15

**Authors:** M. Isabel García-Rodríguez, Lourdes Biedma-Velázquez, Rafael Serrano-Del-Rosal

**Affiliations:** Institute for Advances Social Studies, Spanish National Research Council, Córdoba, Andalusia, Spain; The Open University, UNITED KINGDOM

## Abstract

Pain is an unpleasant experience that we will all experience in some form over the course of our lives, with chronic pain affecting a significant proportion of the global population. Given these circumstances, this study investigates whether pain is a legitimated phenomenon and considers the processes involved in the creation of such a status. This is an exploratory investigation based on semi-structured interviews with people suffering from chronic pain as a consequence of physical, psychological, emotional, or social circumstances. Our principal objective is to explore the fundamental elements of legitimacy and the processes that bring it into being—i.e., to understand how it is socially constructed. The main finding, however, is that many sufferers of chronic physical, psychiatric/psychological, emotional and social pain perceive that their pain is not considered legitimate when no clear cause can be identified, when the pain prevents them from developing the norms imposed by social roles or when it inhibits them to make a productive contribution to the society in which they live. This is generally due to the disruptive aspect of pain and its impact on the social structure, specifically on productivity, which nowadays constitutes a key element in the legitimation of any social phenomenon.

## Introduction

Pain is a growing phenomenon in contemporary societies, and it is something that most people experience at some point in their lives. It has been defined and interpreted in different ways throughout history [1–5] giving rise to a range of classifications [6, 7]. In terms of duration, a distinction is made between acute and chronic pain. Acute pain is according to the IASP “pain of recent onset and probable limited duration. It usually has an identifiable temporal and causal relationship to injury or disease” [8]. For its part, chronic pain commonly persists beyond the time of healing of an injury and frequently there may not be any clearly identifiable cause [8]. These types of pain are very different from each other [9].

In recent decades, paradigms of pain research and treatment have evolved from a biomedical model, focused on treating illness and biological problems, to a biopsychosocial model [10–12] whereby chronic pain and how it is perceived are also mediated by psychological and social factors.

Chronic pain is “an individual, multifactorial experience influenced by culture, previous pain events, beliefs, mood and ability to cope” [13] p. 329, but also by public policy and the sociopolitical context [14]. Furthermore, “pain is a processual concept, under constant review, whose personal and social consequences vary over time” [15] p. 57.

Pain is currently defined by the International Association for the Study of Pain (hereinafter IASP) as “an unpleasant sensory and emotional experience associated with, or resembling that associated with, actual or potential tissue damage” [16] p.1976. It is, therefore, something individual and subjective, which cannot be measured in a precise and objective way, but rather through empirical means based on the experience of the sufferer. However, pain is also an intersubjective experience, since there is a relationship between knowledge about it which individuals have internalized and the social context in which it is produced [17]. This relationship means pain may be recognized even if it has not been experienced first-hand.

In general, the experience of pain is linked to a particular health condition and is understood in relation to this, giving rise to subsequent conceptualizations of pain, according to whether it is associated with a medically recognized illness included in international classifications, or instead with a contested illness. Contested illnesses are conditions in which sufferers and their advocates struggle to have medically unexplainable symptoms recognized in orthodox biomedical terms, despite resistance from medical researchers, practitioners, and institutions [18] p. 153. For illnesses such as endometriosis, central sensitivity syndromes, or fibromyalgia, sufferers have to insist a doctor to be properly heard, to be convincing about their pain, the experiences they have and the results of the treatments [19].

The biological model holds that “only illnesses that are objectively measurable can be legitimated as a disease” [20] p.248. Prior to a situation being considered and classified as a disease, a lengthy process of negotiation takes place between those affected by it and the expert systems charged with its investigation and subsequent recognition.

Indeed, “biomedicine rejects or marginalizes conditions that do not fit into its models of causation” [21]. “If it is not specific, it is not a disease, and a sufferer is not entitled to sympathy” [22] p. 577. Even medically recognized pains such as migraine, lupus, depression, or chronic back pain are also subject to a “perceived illegitimacy as an illness experience” [23] p.1.

The current model for approaching pain does not take into account “structural inequalities that shape who experiences pain, how they experience it, and how others view their pain” [24] p.4 and pain occurring outside the context of a recognized and institutionalized disease is excluded from the social processes of formal legitimation and left at the mercy of other social dynamics. Yet certain symptoms or even the pain itself cannot always be detected by standardized diagnostic medical tests [25]. Indeed, the academic literature contains multiple examples of research into illnesses that cause pain but there is no etiological evidence [22]. Such examples show how difficult it is for sufferers to achieve a degree of social recognition, whether publicly or privately, despite the fact that their pain may be incapacitating. When they lack clarity about the causes, even the spouses of individuals suffering from pain that is uncertain, invisible, or ambiguous interpret the pain they observe according to their pre-existing beliefs [26].

Pain is a medical issue and an everyday experience [27]. The realm of everyday life “is the sphere of reality in which humans can intervene through their living organism and which they can alter, and it is only in this sphere that their fellow humans can be understood” [28] p.25, since this requires the existence of intersubjectivity and of shared values and meanings [29]. So, it seems appropriate to include research from a sociological perspective [24] which allows both levels to be linked.

The legitimacy of pain will be addressed in this article in terms of perception, as the “interaction between the individual and the society they belong to” [30] p. 92.

There has been much research on the concept of legitimacy. In sociology, it was introduced by Weber and Parsons, who understood it as the “congruence of an organization with social laws, norms and values” [31] p.5. Parsons [32] refers to illness as a deviant but involuntary state, in which the sick persons have rights and obligations. They can legitimately exented from working but they have the obligation to do everything possible to get well, as soon as possible. This concept is subject of constant revisit due to the increase in chronic pain, the current meaning of the concept of health and the impact of changes made on productive systems and job insecurity, among others ones.

Legitimacy has gradually been incorporated into research areas such as health research with the idea that context matters [33].

In general, the most helpful conceptualization is that which sees legitimacy as the “generalized perception or assumption that the actions of an entity are desirable, proper, or appropriate within some socially constructed system of norms, values, beliefs, and definitions” [34] p. 574.

New perspectives and elements have been incorporated into the concept. Suddaby [35] distinguishes between three different configurations of legitimacy by asking: What is it? Where does it occur? And how is it produced? These questions offer different research perspectives, and, in this sense, this article approaches legitimacy as perception, focusing on the individual and their interaction with social processes [36, 37].

From the perspective of perception, legitimacy is seen as propriety, in the sense of whether something—pain, for example—is seen as appropriate or not. It can also be defined as a process, constructed through individual judgments [37] but situated nevertheless in the “interaction between individuals’ cognition and supraindividual social processes” [38] cited in [35] p. 463.

Legitimacy has been studied as a dichotomous phenomenon [31], but the need to theoretically clarify the “gray area” between the two extremes of “illegitimacy” and “legitimacy” is increasingly recognized. Three states can be found between these extremes: conditional legitimacy (inside which Osteoarthritis could be located e.g.), unknown legitimacy (inside which Post-viral fatigue syndrome [myalgic encephalomyelitis] could be located e.g.), and conditional illegitimacy (inside which Multiple Chemical Sensibility could be located e.g.) [39].

In terms of the perception of legitimacy and its state, epistemic injustice [40] allows sufferers to become aware of the credibility of their situation in relation to pain. This occurs when “prejudices lead a listener to accord a speaker’s words a diminished degree of credibility” (testimonial injustice) [40] p.17. This also takes place “when a gap in the resources of collective interpretation place someone at an unfair disadvantage when it comes to understanding their social experiences” (hermeneutic injustice) [40] p.17.

The construction of legitimacy also involves specific social processes that do not necessarily affect health. Indeed, a notable feature of our contemporary social order is that health is analyzed and quantified in economic terms [41]. Increasing productivity is one of the central tenets of our contemporary neoliberal economic system. To this end, new models of personnel management have been developed in the corporate sphere before being applied in the public sector too. Workers have had to adapt to elements such as the monitoring of working hours and changing expectations regarding intensity and flexibility [42].

As part of this process, they have to deal with situations created by the system itself, such as time scarcity, the fragmentation of the labor market, and the need to develop new skills in order to remain available to the market and be an active consumer [43].

In this context, workers are been pushed to be as productive as possible, even though it has been observed that pushing their productivity expectations beyond their ability generates discomfort and various pathologies [44]. It is hard to evaluate pain as the exclusive cause of a temporary incapacity, however; it must be considered in relation to everyday actions [45], with employment representing a strategic dimension. Other social processes linked to the economic system such as changes in family structure and size, the move toward individualization [46], or the increase in individual responsibility for life, health, and pain experienced. The common denominator in all these situations is that the individual must be productive, and one of the greatest obstacles to this condition is pain.

In sum, this article analyzes legitimacy of pain from de perspective of sufferers themselves. The theoretical perspective that is used here is the biopsychosocial model of pain and the theory of legitimacy as perception and as a process. A sociological analysis of the legitimacy of chronic pain is presented.

## Materials and methods

For the qualitative research, a series of semi-structured interviews were conducted with sufferers of pain of different origins. Fieldwork—including sample preparation, script design, participant recruitment, pre-testing, and interviewing—lasted from October 2019 to October 2020.

Purposive sampling [47] was used in order to ensure that the group of participants was as heterogeneous as possible. The criteria used for sample composition were as follows: 1) the origin of the pain. Interviewees suffering physical pain, defined as a subjective sensation of unpleasantness in a part of the body, caused by organic mechanisms provoked by lesions or disfunctions, with an enormous variability of causes. Interviewees suffering physical pain, which may be nociceptive or neuropathic [48]. Interviewees suffering psychological pain, defined in general terms as “a diffuse subjective experience,” “differentiated from physical pain” [49] p.681 and “associated with major psychiatric disorders” [49] p. 681. Interviewees suffering emotional pain, defined as a type of psychological pain that occurs as an internal response to severe social stressors [49] p. 681. Interviewees suffering social pain, defined as pain suffered when experiencing a negative social situation, like bullying, isolation, or social exclusion, provoking an emotional response of pain [50]; 2) inclusion of different types of pain within each origin category; 3) inclusion of men and women; 4) inclusion of a wide range of ages (minors under 18 are excluded); 5) geographical representation (distribution by autonomous community); and 6) inclusion of both pain whose origin is known and pain that is not yet recognized or is biologically invisible or is linked to a contested illness [51, 52]. See Table 1.

**Table 1:** Sample. Origin of the pain. Pain. Identifiers of pain. Means of communication. Autonomous community.

	Pain	Identifiers of pain	Means of communication	Autonomous community
**Origin of the pain**		ICD-11 CODES*		
Physical	Osteoarthritis	FA0Z	In person	Madrid
	Chronic migraine	8A80	Telephone	Andalusia
	Obesity	5B81	In person	Andalusia
	Generalized pain [fibromyalgia]	M630.01	In person	Andalusia
	Lupus	4A40	In person	Valencian Community
	Post-viral fatigue syndrome [myalgic encephalomyelitis]	8E49	Online	Castilla La Mancha
Psychiatric/psychological	Schizophrenia [schizoaffective disorder]	6A20	In person	Andalusia
	Borderline personality disorder	6D10.Z	In person	Andalusia
	Chronic depression	MB245	Telephone	Extremadura
	Gambling addiction	6C50.Z	In person	Andalusia
		ACRONYMS**		
Emotional impact	Daughter’s death	EIDD	In person	Andalusia
	Wife’s death	EIDW	In person	Andalusia
	Relationship breakdown	EIRB	Online	Catalonia
	Rejection due to sexual orientation	EIRSO	Telephone	Andalusia
Complex social situations	Chronic fatigue syndrome	CSSCFS	In person	Andalusia
	Comorbidities. MCS. ES	CSSCM	Online	Madrid
	Gender-based violence	CSSGBV	In person	Andalusia
	Bullying	CSSB	In person	Andalusia
	Owing to adoption	CSSA	In person	Andalusia

Source: Compiled by the author

*Pain listed in the ICD-11 classification. Henceforth referred to by their ICD-11 code.

**Pain not classified in the ICD. Henceforth referred to by their acronym.

A dual strategy was employed to gain access to the field. First, we contacted various associations for the illnesses or situations associated with each pain to ask for their collaboration, specifying that the person interviewed must be a sufferer of pain regardless of their position in the organization. To contact harder-to-reach participant categories, we hired a company specialized in field work.

In total, nineteen interviews were carried out, one for each type of pain identified, distributed throughout various autonomous communities in Spain. See Table 1.

Interviews were carried out in a single stage, but diversity in the types of pain included in the sample and the number of interviews carried out made it possible to achieve saturation of the field, that is, “when no new categories or relevant themes are emerging” [53] p. 39.

### Data collection

The interviews were conducted by a man and a woman from the research team with training and experience in qualitative research and fieldwork.

The interview script was designed in line with the research objectives and was tested during the initial phases of the fieldwork. Its final structure comprised eight sections exploring the construction of the legitimacy of pain. These are as follows: 1) current situation; 2) onset and development of pain; 3) description and behavior of pain; 4) coping with pain; 5) diagnosis; 6) impact of pain on the work, family, and social spheres; 7) social attitudes to pain; and 8) types of pain that are legitimated to a greater or lesser degree.

The interviews were adapted to the cultural and linguistic level of each participant. The following means of communication were used: in person (13), via the internet (3), and by telephone (3). We had hoped to carry them all out in person, but the lockdown introduced during the COVID-19 pandemic rendered this impossible. Thus, the remaining interviews were conducted using the most appropriate technology for each interviewee. See Table 1.

All interviews were recorded and later transcribed. The average interview length was 80’50”. The sociodemographic characteristics of the sample are shown in Table 2 below.

**Table 2:** Sociodemographic characteristics of the sample.

Sociodemographic characteristics N = 19		Pain of physical origin N = 6 [31.6%]	Pain of psychological origin N = 4 [21.1%]	Pain of emotional origin N = 4 [21.1%]	Pain arising from complex social situations N = 5 [26.3%]
Age	Average, [range], years	55 [29–84]	41.8 [35–50]	52.5 [44–56]	48.2 [42–56]
No. of Children	Average, [range]	1.3 [1–4]	2 [0–2]	1.5 [0.2]	2 [2]
Sex	Male	1	2	4	1
	Female	5	2	1	4
Level of education	Primary	1	-	-	-
	Secondary	-	1	-	-
	Vocational Studies	1	1	-	1
	University	4	2	4	4
Marital status	Single	2	2	1	-
	Married/in a couple	2	-	1	4
	Divorced/separated	-	2	1	1
	Widower/widow	1	-	1	-
Size of community	<20,000	-	-	-	1
	20,001–100,000	2	1	1	-
	>100,001	4	3	3	4
Employment status	Employed	3	3	4	4
	Unemployed	-	1	-	1
	Retired/on sick leave	2	-	-	-
	Housework	1	-	-	-
Occupation	Skilled	4	2	4	4
	Unskilled	2	2	-	1
Member of an association	Yes	4	4	2	2
	No	2	2	2	3
Total age	Average, [range], years		49.89 [29–84]		
Total no. of children	Average, [range]		1.37 [0–4]		

Source: Compiled by the author

All interviewees participated voluntarily and were informed of the research objectives, the voluntary nature of the interview, and that it was going to be digitally recorded. They were informed of compliance with Organic Law 3/2018, of December 5, on the Protection of Personal Data and guarantee of digital rights. Informed consent was obtained from all subjects involved in the study. They signed an informed consent document approved by the ethics committee. The research has obtained the certificate of the Research Ethics Committee of Córdoba on January 28, 2020 [Act No. 299]

### Analysis

Qualitative content analysis “is a research technique for developing inferences to systematically and objectively identify certain specific characteristics within a text” [54] p.5. Broadly, it allows the analysis of phenomena that cannot be directly observed through interview transcripts. The analysis was carried out using twelve codes, organized into three categories at the end of the analysis. The system of codes gave rise to a group of theoretical categories, derived from a systematic analysis of the data, allowing the data to be interpreted and a theory to be developed [55]. The process was carried out by means of the reduction and fragmentation of texts. All sections of the text that were relevant to the research objectives were assigned a code, and the codes were then grouped into broader categories.

An inductive-deductive procedure was carried out. First, the texts were read to identify and code themes of interest, also drawing on prior knowledge obtained in previous studies by the research team, and from other studies. Legitimacy States in the gray zone [39] are included among them.

A range of actions were taken to ensure the quality of the analysis. First, the COREQ (consolidated criteria for reporting qualitative research) interview checklist was completed and is attached as S1 File. The coding tree is attached as S2 File, and the interview script is attached as S3 File.

The following measures were taken to avoid possible biases in the coding and categorization process: coding of the interviews by two members of the research team; exposition of the coding to all team members to detect weak or irrelevant relationships. Members of the research team checked and provided a critical perspective, and they assessed the decisions made during data analysis [56]. The processes of coding, categorization, and verification of the suitability of the verbatims were checked. A dynamic discussion of quality issues also took place among the research team [57].

## Results

Sufferers of pain do not feel that their pain is legitimated; on the contrary, they feel that as a social situation their pain undergoes a constant process of evaluation which manifests as sufferers interact with the various components of their daily life. Each sufferer claims to have experienced a specific process of the questioning of pain, although their narratives also have general aspects in common with other sufferers. All of them seek to achieve a certain degree of legitimacy.

We will first consider the social processes involved in the formation of legitimacy or illegitimacy of pain from the perspective of those affected. Second, we explore whether their pain is legitimated by society. Finally, we present the basic rules that have to be obeyed in order for pain to be as legitimate as possible.

### Social processes involved in the lack of legitimacy of pain

Individuals reflect on their pain, its intensity, duration, impact, and many other issues related to it. In this reflective process, they analyze how their pain is seen within the framework of their social relations, be that in the public/institutional sphere or in daily life. The perception of pain itself tends to be situated on a continuum between legitimacy at one end and illegitimacy at the other. There is a gray area between the two extremes in which different “states of legitimacy” are located [39].

Three main relational processes are involved in the evaluation of the state of legitimacy of pain. The first involves the relationship of sufferers with expert systems [58]. The second is found in the way sufferers relate to the norms of the “social fields” [59] of which they form part, particularly the field of work. The third is seen in communicative and testimonial situations involving sufferers, in the form of epistemic injustices [40].

#### The relational process between sufferers and expert systems

Expert systems function as distributive sources of legitimacy, given their role as mediators between citizens and knowledge [60]. Via formal elements such as diagnoses, assessment procedures, tests, certifications, or verdicts, they objectify pain, validating its existence.

Given the institutional design of the Mediterranean welfare state [61] characteristic of Spain, the health system is one of the most important legitimacy awarding entities, since it attests to the health of citizens. Its main way of approaching pain is to objectify it via the medical tests used to arrive at a diagnosis. A diagnosis affirms the existence and veracity of the pain and makes the sufferer’s everyday experience comprehensible.

This normative legitimacy [31] opens the door to other public resources: treatments within the health system itself, as well as the disability and incapacity-for-work benefits dispensed by other social protection systems.

However, the establishment of a diagnosis is often subject to interference caused by the system itself. Long waits for tests or appointments with specialist doctors, a lack of knowledge about pain and certain conditions or their insufficient institutionalization, and even the way services are organized all have consequences for pain. In all cases, legitimacy is subject to debate between the system and sufferers. Normative legitimacy is achieved more easily by those whose pain is medically recognized, although they are also faced with processes of negotiation within a state of unknown legitimacy. In other words, pain must be scrutinized and agreement reached between those who are assessing it [39].

Some factors that contribute to legitimacy remaining in an intermediate state of lack of knowledge are: the absence of a diagnosis [8E49]; receiving a late diagnosis [4A40]; receiving erratic diagnoses [CSSCFS]; contradictions between scientific discourse and medical practice [5B81]; and the undermining of the importance of intersubjective understandings of pain [FA0Z]. See Box 1.

**Box 1**

[…] a feature common to both MCS and, to an even greater extent, CFS, is under-diagnosis [8E49]

[…] I was told it was a temporary arthritis. That in time it would resolve itself. Well, in time what actually happened was that it attacked my kidney […] [4A40]

[…] the internist tells me that [. . .] it’s a big jumble [CSSCFS]

[…] the strangest thing is that the doctors […] tell you that if you don’t have diabetes, high blood pressure, or high cholesterol, then you’re metabolically healthy. You’re a healthy kind of obese […] But that’s like saying you have cancer, but you’re healthy [5B81]

[…] they know it hurts because a nerve rubbing on bone is extremely painful. But they often say, “take a painkiller because it’s a long process,” that sometimes it’s two months before you get to traumatology, then they refer you to get a […] scan and then they refer you to the neurosurgeon, who is the person who… and then they finally give you a date for the operation. [FA0Z]

Another factor that inhibits the legitimation of pain in the health system is that of failing to provide sufferers with information about pain or not providing this information correctly, such that sufferers are deprived of knowledge about their situation and find themselves in a position of helplessness. See Box 2.

**Box 2**

When they gave me the diagnosis it was liberating to know what I had, but at the same time, […] why didn’t they tell me eight years ago? [6D10.Z]

[…] a doctor in a press conference who said “osteoporosis doesn’t hurt” [FA0Z]

[…] this is mainly because, at least for women, it’s less the case now, but […] I’ve even had a neurologist tell me: “the problem is that you’re depressed” [8A80]

The first thing is that they don’t recognize you as such […] I go to the family doctor […] and they always talk about being overweight, overweight, overweight, and being overweight is not obesity [5B81]

So, nobody could diagnose it. I wasn’t diagnosed until 2016 […] because there hasn’t been enough research […] It sounds like absolute gibberish to general practitioners [CSS.MCS.ES]

From the participants’ accounts, it is clear that sufferers of pain are sometimes given psychiatric diagnoses, regardless of whether their pain matches an already-known disease. Indeed, this happens in the case of migraine pain, which is relatively well known within the health system, as well as fibromyalgia. Sufferers do not receive adequate care and are treated with suspicion rather than any kind of recognition that pain can manifest itself in a variety of ways, regardless of the presence or otherwise of any material injury [M630.01; 8A80; CSSCM]. See Box 3.

**Box 3**

“Your problem is that you’re depressed” […] I haven’t been depressed in my life [M630.01]

I’ve even had a neurologist tell me: “the problem is that you’re depressed” [8A80]

And this doctor says to my sister: “gee, I feel awful for thinking that that girl who came in wearing a face mask was crazy” [CSSMCS]

Biologically invisible illnesses [62] and others that are insufficiently institutionalized, such as MCS, are in the realm of unknown legitimacy, with little legitimacy, which can cause great pain. In the case of socially contested illnesses [18], the reaction is so intense as to call into question the very existence of the illness, as in the case of the pain experienced by those suffering from electro sensitivity or MCS. This pain is in a space of illegitimacy conditioned by the wait for some kind of validation, be that practical in the form of the application of effective pain relief, or normative through it being recognized as an illness and classified as such in international diagnostic manuals. All those pains are potentially incapacitating in terms of everyday life, and working life in particular, whether temporarily or permanently.

Such illnesses frequently do not obtain normative legitimacy in the health system, nor in the systems that give rise to some forms of pain, such as the education system in the case of bullying, for example, making it necessary to resort to the judicial system in order to have the pain and its impacts on the sufferer recognized by the Instituto Nacional de Seguridad Social (INSS) (National Social Security Institute). See Box Box 4.

**Box 4**

If you’re working […] you have to get signed off. The INSS will discharge you and send you back to work, because that’s how they operate with this kind of illness [8E49]

It’s very hard. [Does it require a lot of resources?] A great deal, on all levels [8E49]

[The mother of a victim of bullying who didn’t receive the attention she had hoped for at school] I’d already been to the school about this, not once but hundreds of times, for discussions [CSSB]

[the school psychologist] didn’t do anything for my daughter when she needed it [CSSB]

Court cases tend to be lengthy and expensive for most of the population who need them. When sufferers are seeking to gain moral legitimation of their situation, seeking recognition of their honesty, the economic and emotional costs of doing so act as a deterrent, not to mention the time this can take [CSSB]. See Box 5.

**Box 5**

[Advice from a psychiatrist] “Is your daughter happy now? […] Don’t carry on. Don’t go through with it, because you’re in for another year […] They’ll put you face-to-face […] You’ll have to undergo a load of psychiatric tests […] They’ll find her […] And I don’t think it’s worth it, unless you want the money.” Response: I’m like, from them? I said, “I don’t want a thing.” They said, “well keep going to therapy then,” and that’s what I did [CSSB]

Emotional pain—such as that experienced by adopted minors or the pain caused by bullying—requires the education system to act as a source of public legitimacy. Yet the system does not usually intervene to prevent situations that cause pain, at times ignoring them and at others acting inefficiently. See Box 6.

**Box 6**

[…] they told me, “sure, something’s going on, but it’s just what little girls do.” They’ve seen something, but they can’t prove it [CSSB]

[…] my daughter said she wanted to die, […] and they told me that maybe she was exaggerating [CSSB]

Those affected point out that the system is concerned with maintaining results for the student body as a whole and does not take into account specific cognitive and emotional needs, such as those of adopted minors, who thus experience additional pain as a result. See Box 7.

**Box 7**

[. . .] I don’t want my children to suffer more because of being at school. That is another pain […] we expose them to when they are here. The pain of dealing with […] academic failure, having the […] bar set so high [CSSA]

Other inhibiting factors of legitimacy of pain that have been identified in the education system include: rejection, stigma, mockery, and cover-ups, as shown by tolerance of the abuse of minors with different sexual orientations, which have led to illnesses and chronic pain as adults. See Box 8.

**Box 8**

[…] when I went to school, [. . .] it was like going to the slaughterhouse [EIRSO] […] They would order me up to the blackboard or ask me things in class to get me to speak, and everyone would laugh at me [EIRSO]

For me, my childhood and teens, up to the end of eighth grade […] were […] were a nightmare. For me it wasn’t a nightmare being gay, but the situation at school […] was devastating because between them they destroyed me. They totally destroyed my childhood, my teens […] the childhood I had was horrible, horrible, awful. It has […] left scars, wounds, some of which have healed, but others haven’t [EIRSO].

Lastly, the participants’ accounts show that diagnosis is a necessary but not sufficient condition in order for pain to reach a state of legitimacy, indeed, even where a clear and precise diagnosis exists, legitimacy can still be lacking. For example, the various types of pain resulting from gender-based violence, lupus, osteoarthritis, migraines, or borderline personality disorder are all still subject to doubts over their veracity. Some are in a state of unknown legitimacy, and others in a state of conditional illegitimacy.

The case of pain caused by gender-based violence is paradigmatic because, despite being situated at the legitimacy end of the continuum and holding normative legitimacy, it coexists with an assessment of illegitimacy in the family and social spheres. Legitimacy is not granted because the legitimizing element in these spheres is the locus of control, the will of the sufferer. The sufferer has not obeyed the norms and social roles of the sex/gender system (she has divorced, left her children with their father, and has had other partners). Furthermore, she has had relationships with men who have turned out to be violent, and currently lives with one such man “of her own free will.”

#### The relational process between sufferers and the field of work

In all the narratives on pain, the field of work is the most influential field when it comes to settling the issue of the legitimacy or otherwise of pain, because all labor activity is productive. A sufferer’s employment situation and consequent living standards will depend on the social qualification of their pain. See Box 9.

**Box 9**

[…] even the Civil Guard advised me not to withdraw it [the complaint] again. And my mum didn’t want to take me in […] saying that she doesn’t want to know anything about it, or about my life. [Her mother said] “you are the one who’s putting up with it all. We can’t do anything” [CSSGBV]

[Friends say] “but hey, you’re an INSULT, and you’ve gone from dumb to INSULT, and we don’t know any more how bad you’ve got, because you’re getting worse.” [CSSGBV]

[About her friends]

Ask. Do you think they’re right? That you shouldn’t be with that guy?

Answer. This is the problem. I do think they’re right

Ask. Sure

Answer. That I don’t want to accept it. I’m really dependent on him. [CSSGBV]

Social fields are regulated by operational rules with which individuals must comply. In the field of work, the main rule is that of achieving maximum productivity and complying with the organization of the company. The legitimacy of pain is always inhibited in this sphere regardless of its legitimacy in other spheres, when the sufferer interferes with the fulfillment of either of these rules.

Individuals have internalized the idea that productivity in their workplace depends on them and not on a broad set of economic, political, and social factors. In this context, responsibility for pain has been individualized to the point that companies sometimes label sufferers as non-compliant or negligent, casting doubt on the reality of their pain. See Box 10.

**Box 10**

Because my own […] director, my own bosses, my own directors didn’t understand. They said: “but let’s see, does it hurt that much? Today as well?” Of course, I understand that it was a problem for them that I missed work, but if I miss work one day it’s because that day it was so intolerable that it was impossible, but they didn’t understand at all. They didn’t understand, and they work in health care. I mean […] can you imagine […] can you imagine what it’s like when this happens to someone in a different job? [8A80].

Sufferers can even feel guilty about the situation in which they find themselves. The most serious consequence of pain affecting performance at work is dismissal [CSSGBV], which is more prevalent in private companies [CSSCFS]. However, other circumstances, such as not replacing workers who are suffering with pain, create an overload for other workers [M630.01], who end up questioning the legitimacy of such pain. There is an underlying and generalized suspicion that workers are dishonest [FA0Z; EIDD] or that they seek to obtain preferential treatment [4A40]. This atmosphere of suspicion also arises among workers themselves, and it can only be neutralized, to a degree, when the sufferer has a proven track record of being efficient and effective in their job [M630.01]. See Box 11.

**Box 11**

[…] “does it really hurt that much? Still?” […] I get that it was a problem for them if I was off work [8A80]

I lost my job because one time I shut myself in my flat […] because I’d been beaten […] I had been on a permanent contract [CSSGBV]

I didn’t lose my job […] If I’d been working in the private sector it would have been more problematic [CSSCFS]

[Of a coworker] I support her one hundred percent, but they have to replace her, otherwise we have to take on all her work on top of our own [M630.01]

[When a person does hard work without saying that he is in pain, the day he stops working for this reason, his behavior is interpreted as voluntary] [. . .] “you’re overreacting. If you’ve managed to do the work so far, surely you can carry on doing it” [FA0Z].

Having to go every two weeks to get signed off work, […] they didn’t let him grieve […] He was really poorly treated [EIDD]

When it comes to work, nobody understands you […] If I say anything, everyone will be like: “look at the teacher’s pet” [4A40]

I think I really make up for it when I’m there, because I work a ton […] Since I already had a good reputation for being professional. . . I haven’t had many problems, you know? [M630.01]

It is certainly true that the social structure itself can compel the most vulnerable workers to behave fraudulently when circumstances such as caring for the elderly or for dependent minors come into play. The lack of social policies and the privatization of these services combine to put individuals in the impossible situation of risking their job or meeting their family’s needs [FA0Z]. The less well-off are harder hit by these circumstances. See Box 12.

**Box 12**

Because if you have a child, [. . .] who stays at home? Who takes them to the doctor? If the mother works, she has to ask for permission, and if [. . .] they don’t give it to you, you have to pretend you’re sick [FA0Z]

And with regard to finding work, it’s not great. I can’t find a job. I’ve been caring for elderly people. But off the books [CSSGBV]

When the right conditions are in place for treating pain, such as access to health care, stable employment, and incapacity benefits, a sense of privilege arises. Equating the existence and respect of social rights with privilege could be considered a proxy for understanding the extent to which neoliberal and individualistic socioeconomic values have penetrated the field of work, including among workers themselves [M630.01; 8E49; EIDD]. Box 13.

**Box 13**

I feel very privileged […] I have savings, an office I can shut if necessary without major problems, and […] I mean, I’m in a privileged position […] [M630.01]

I’m a civil servant! And very proud of it. So my situation has been very privileged [8E49

[…] I am lucky in that my company is a multinational […] My boss told me […] come back, just start with what you can manage […] Yes, I felt supported [EIDD].

In the best-case scenario, the legitimacy of pain is conditional on the sufferer meeting expectations in terms of productivity. If they do not meet these due to their pain, whether it is medically verifiable or not, sufferers will face a “trial” [37], which prevents the legitimacy of the pain being wholly established. Sufferers cannot gain legitimacy that would allow them to resolve their problems, because pain interferes with the company’s economic goals.

In fact, the legitimacy of pain is perceived as a cost that the state cannot allow and which should be reduced by increasing resources for, and research on, correct treatment. See Box 14.

**Box 14**

“How can it be that such a serious illness, with such an impact, and which is costing the state so much, is not being investigated or noticed?” [8E49]

The relational process in communicative and testimonial situations among sufferers when these involve epistemic injustices

Here we refer to sufferers’ experiences of being doubted in communicative situations when they speak about their pain. Their interlocutors may employ a range of responses, such as downplaying the importance of the pain communicated, doubting the veracity of what has been said, denying the pain, or making erroneous interpretations, as they lack the necessary knowledge to understand the pain.

Sometimes, injustice occurs in the field of medicine when pain is denied [FA0Z], in contradiction of the IASP’s definition. Sometimes pain is downplayed by reducing it to a normal consequence of activity like tiredness [M630.01]. In other social contexts, testimonial injustices are committed by disregarding the sufferer [6D10.Z], claiming they are pretending [EIDD], or denying the reality of their experience [CSSB]. See Box 15.

**Box 15**

[…] at a press conference, a doctor said, “osteoporosis does not hurt,” and when the media had finished recording, I said to him, “don’t ever say that again when I’m around” [FA0Z]

“What you need to do is to rest [. . .] because you work a lot and of course that. . .” but without understanding that it is an illness [M630.01]

And it gets to the point of, “okay, you clearly don’t understand me.” At the end of the day, if I’m explaining how I feel and you’re poking fun at me, there’s not much more to say [6D10.Z]

So, they see you at a party: “Look who’s got depression”; “Hey! Just look how depressed she is.” No, she just needs to do this, okay? [EIDD]

[Mother of a victim of bullying] I just couldn’t believe it at first […] because it was just so far-fetched, so hard to believe, that my reaction was: “this can’t be true. Either she’s got a problem and so she’s playing it up for some other reason, or this can’t be true” [CSSB]

Testimonial injustice contributes to impeding the legitimation of pain, be it that by considering it in a state of conditional legitimacy, that is conditioned to complying with certain norms relating to appearance or behavior, or by considering it in a state of unknown legitimacy. Sufferers, then have to prove that their pain is real and that they are trustworthy citizens by showing or referring to different ways of objectifying it. Nothing protects those in pain from hermeneutic injustice, however. Hermeneutic injustice comes about when neither society nor expert systems have the necessary knowledge to recognize the idiosyncratic nature of a particular pain—that of myalgic encephalomyelitis, MCS, ES, or the ever-present pain caused by complex social situations such as gender-based violence, for example.

### Evaluation of the legitimacy of pain according to sufferers

Interviewees perceive their pain as not having the accepted status of full legitimacy that it should have. They see a series of circumstances that impede their social acceptance as people who are truthful and credible in their experience of pain. That is to say, no pain is judged by society as completely legitimate.

Pain can be understood intersubjectively, but it cannot be experienced vicariously, and this is a key impediment to pain being legitimated, recognized, dealt with, and respected in all spheres of life. Reflections on pain itself show that elements such as not having the appearance or behavior expected of a sufferer, arousing suspicions of fraudulent behavior to obtain undue benefits (malingering), the creation of fear of others because of what is being experienced, or the lack of societal understanding of certain kinds of pain or disruptive situations are factors that make a pain more or less legitimate Table 3.

**Table 3:** Perception of the legitimacy of pain according to one’s own.

Most Legitimized or Most Respected Pain	Less Legitimized or Less Respected Pain
**Individuals Suffering Physical Pain**	
Everyone can see it Breakage of a limb	There are no objective tests to confirm them. Pain that cannot be explained Mental pain
They can be demonstrated Pains endorsed by medical test Cancer Death of your own child	This cannot be confirmed with evidence Migraine Fibromyalgia
Pain from external causes (external locus of control) Pain by organic causes (Pain or illness improves or disappears) Diabetes	Responsibility for pain (internal locus of control) Lack of willingness Obesity
Everyone can see it They can be demonstrated Pain that allows you to work Pain from a broken limb	That one that cannot be proved Griefs Mental pains Mental ailments Emotional discomfort
Pains accepted by medicine Pain from a broken limb Death of a loved one Family breakup	Those whose manifestations are unseen That one that cannot be proved
**Individuals Suffering Psychological Pain**	
Pain symptoms are not noticeable. The pain allows to fit in Cancer	That one whose symptoms are noticeable The one that does not allow to fit in
The pain is seen To have an arm in a cast	The pain that is not seen Being an internal pain
No pain is legitimized. People are cruel	psychological pain
That pain is considered a disease. Cancer	When pain is considered a vice Addictions before being conceptualized as diseases
**Individuals Suffering Emotional Pain**	
Pain allows one to be useful to society	Those that may seem as malingering
Pain allows one to join family life	Those who make participating in family activities difficult
Pain caused by deadly diseases Sorrows caused by illness of children The pain of your own death Leaving children alone after death	Loss of partner due to divorce
It’s a physical pain The pain can be verified with medical tests	The ones that cannot be quantified Psychic pain. psychological pain
**Individuals Suffering Social Pain**	
Pains accepted by medicine	Those that interfere with the performance of social roles
Pain by major disease The pains that can be seen Pain by cancer. Having screws in one leg from an operation	Those with unusual symptoms
Pain that is not considered pain	Those one that are little known Those that cause family rejection
Pain that you see Maltreatment	Those who lack credibility. Those whose tests are not accepted. Psychological abuse
Doing your part to receive help.	When others think you can control the situation but you can’t.

Source: Compiled by the author

In terms of the legitimacy of pain in general, the interviewees draw a distinction between different kinds of pain using a series of criteria. The most legitimate are those that are most visible to society, those that can be confirmed from a medical-scientific perspective, those that do not interfere with the fulfillment of social roles, and those that are accepted as completely legitimate in social discourse, such as cancer, diabetes, sclerosis, or the fracture of a limb. The norms, values, and beliefs underlying how pain is considered are quite homogeneous, and they do not vary significantly according to its origin.

Sufferers perceive that there are social judgments that prevent pain from having a legitimacy status. They are listed in Table 4.

**Table 4:** Elements that prevent the social legitimation of pain. Sufferer’s Perception.

**Individuals Suffering Physical Pain**	
Social unawareness of pain	Attributing the pain to sex-gender. Women exaggerate, they are hysterical.
Insufficient scientific evidence	Pain cannot be endorsed with objective evidence
The one who cannot be seen is more questioned	Internal locus of control. Individual responsibility for pain.
The behavior of the sufferer does not correspond to the social expectation	Unwillingness
The appearance of the sufferer does not correspond to the social expectation	Malingering
Attribute the pain to fatigue	There is no vicarious experience of pain.
Attributing physical or organic pain to mental health	Not wanting to experience what pain means to those who experience it
Attributing pain solely to age	Because pain interferes in the lives of others
If pain interferes with work (doubts arise about its veracity, intensity, etc.)	Sufferers are lazy. They don’t want to work. The pain comes from a vice (addictions)
Failure to comply with social gender roles	
**Individuals Suffering Psychological Pain**	
The pain is not seen	Internal locus of control
Not having problems that society has labeled as important (poor living conditions, not having a family, loneliness. . .)	Responsibility for pain
To be scary	Not being able to organize adult life
That the disease traits are visible	Suspicion of simulation. Want a sick leave
Low empathy	Stigma
Don’t fit in	Lazy. Weak. Lazy
Disbelief about pain	Dehumanization: the individual is the disease
Being unable to work	Not complying with gender roles
Inability to have a social life	
**Individuals Suffering Emotional Pain**	
Not to be useful. Don’t function in the economic system	Pain is trivialized because it does not have an organic origin
Not conforming to social norms	Pain minimization
The pain they feel scares others	If you have what society considers important (work, children, home) pain is not so important
Do everyday life (as if nothing had happened)	Self-destruction
Blame the sufferer	Not accepting that it is a pain like the others
There is no vicarious pain	Not being able to work. Suspected fraud
Failure to follow family rules	The pain must be overcome in a certain time
**Individuals Suffering Social Pain**	
Pain is not understood	The pain is not seen
Internal locus of control Individual responsibility You are lazy	Pain and symptoms confront common knowledge
Failure to fulfill social gender roles (Fatherhood and motherhood)	The word of the mourner alone is not enough
Not look sick. Have an appetite	Vicarious experience does not exist
Have a social life with friends	Not complying with social norms. Craziness
Attribution of pain to a mental problem	Believe that pain does not exist
Having symptoms that are attributed to recognized illnesses	Being subjected to a high demand of performance without considering the pain
They do not believe you	Not being “of the same blood” Minors in adoption situation
Not being able to get out of a complex situation	

Source: Compiled by the author

### Strategies for legitimating pain

Legitimacy is not an inherent characteristic of pain. Rather, it should be understood as the unlikely result of a set of social relations. Pain can be said to be legitimated when it is correctly diagnosed and treated, and when it is known, recognized, and accepted by society. In the narratives, this is envisaged as the moment that a specific type of pain reaches the same status as other types that are already considered legitimate [5B81]. See Box 16.

**Box 16**

I’ll give an example […], because it is the one people are most familiar with: diabetes. You can’t control it because it’s physical […] Well, obesity also has that physical aspect [5B81]

The narratives show that only very rarely do sufferers feel their pain is legitimated, given the reactions it provokes in their social relations. Conscious of the position their pain places them in, whether one of conditional legitimacy, unknown legitimacy, or conditional illegitimacy [39], they all develop strategies to legitimate their particular situations, although these do not tend to be very successful.

Of primary importance is the search for a diagnosis, whether in the public or private healthcare system, since this represents a necessary condition for obtaining legitimacy, but this is not enough, in spite of everything. Diagnosed sufferers continue to be suspected of making it up or pretending in other social fields and in their personal relationships. The most frequent situation is that these pains remain in a state of conditional legitimacy. In this sense, the fact that pain cannot be experienced vicariously represents a stumbling block [6D10.Z], as shown by the fact that being close to pain or having experienced it oneself facilitates its understanding and legitimation. See Box 17.

**Box 17**

Because they don’t know what it’s like on the inside, that it’s very bad. I really mean it—it’s terrible [6D10.Z]

It took me four years […] having to go up in front of the district director with a series of documents from all the neurologists and say: “look, here is my medical history. You can see that I’m not absent because I want to be. Look, look at it. As a doctor, look at it. As a doctor, look at it.” For me it’s been… it’s caused me huge moral distress [8A80]

Certain elements of what is known as the “sick role” [32] remain in force (such as the visibility of pain or its impacts) and are necessary in order for a pain to be considered legitimate. See Box 18.

**Box 18**

Of course, when you’ve broken a limb—your leg or arm or wrist—everyone can see it [FA0Z]

However, sufferers are also required to be prudent. Not talking too much about the pain increases its legitimacy because it allows the sufferer to maintain the appearance of mental stability and self-control [M630.01]. Keeping quiet about pain helps to preserve the dynamics of the social environment by protecting it from potentially disruptive situations. It is necessary for sufferers to exercise self-control when speaking about their pain in order to avoid creating the impression that they need more attention than other members of the group; were this to occur, the group would find it bothersome and tedious [8E49]. See Box 19.

**Box 19**

Even the physios treat me as if I were half-crazy, you know? Yes, “see a psychiatrist,” one told me. “I can’t help you—go and see a psychiatrist,” because when he touched me I would cry [M630.01]

If you’re always talking about what you’re going through, people get fed up of you […] [8E49]

In general, some degree of legitimacy is sought by attributing to the pain a physical or biological causality or nature that is beyond the sufferer’s control or will. This absolves the sufferer of any responsibility without calling their mental health into question, thus protecting them from suspicion [6C50.Z]. See Box 20.

**Box 20**

With gamblers, our brain secretes a kind of substance—I can’t remember its name right now, but that’s what makes us gamble [6C50.Z]

Yet, as sufferers show, this strategy is not always successful. Let us take as an example the impossible legitimacy of obesity. Obesity produces musculoskeletal and other types of pain, is visible, and has been conceptualized as a health problem. However, recognition tends to be lacking for those with lower levels of morbidity. Meanwhile, those whose morbidity is more pronounced are often seen as having little self-control and failing to take responsibility for their situation. They find themselves continually under suspicion, such that legitimacy or illegitimacy is built around the evaluation of occasional behavior rather than the physical, psychological, or emotional conditions that give rise to the pain. See Box 21.

**Box 21**

[We’re subjected to] social pressure, pressure from our families […] if we’re on a diet because we’re already overweight. We’re highly visible, so we get pressure from all sides […] [5B81] […] We’re always being watched and questioned [5B81]

At work, the main strategy is to preserve the legitimacy that comes with being a productive and efficient worker who complies with company policies.

Attempting to sustain the level of activity one had prior to the onset of pain is a common strategy for defending oneself against possible attacks from one’s environment. These may be directed at the performance of social roles, such as being a good father, a tireless worker, a competitive sports partner, or a friend who participates actively in social gatherings. Pain does not have the slightest legitimacy until its consequences are visible and so intense that they cannot be ignored [CSSCFS]. See Box 22.

**Box 22**

[…] my wife too, who used to say, “what’s wrong with you?” and that kind of thing […] [She thought] he’s always been very active and now he’s forever lying down. “What’s the matter with you? Don’t be silly.” But in the end, she saw what was going on, and it doesn’t bother me anymore [CSSCFS]

It’s so intense, that it. . . The pain is so immense that it stops you from doing anything at all. It affects everything, your entire organism […] My eldest daughter says, “you’ve got a really bad headache. You should go and lie down” [M630.01]

The strategies mentioned are individual, but pain can also be legitimated collectively in certain circumstances. Campaigns to raise social awareness are very helpful as they achieve recognition for sufferers [CSSCM]. It can also help to know a person who is suffering from pain and share spaces with them [CSSCM], because this makes it possible to observe the role played by context and the practices effected while pain is present. Lastly, mobilizing sufferers via associations and awareness or fundraising campaigns tends to bring about a high degree of legitimacy for their pain [8E49]. See Box 23.

**Box 23**

We thought she was crazy, and now that we’ve seen what happened to you, I feel guilty for thinking that about the other girl [CSSCM]

I noticed that at first nobody understood me, and now, perhaps since she [her sister] has started [The interviewee has gained legitimacy since her sister feels the same pain, because she has more credibility among her family and friends.] [CSSCM]

We have collaborated with some studies as an association [. . .] We have also collaborated as a company. I’m telling you, 15% of what we got from a solidarity fundraiser […]—there you go, mister, for your research [8E49]

Legitimacy cannot occur without recognition, but recognition also requires a degree of legitimacy, such that both conditions must be met. It is a chicken and egg situation. Indeed, when seeking legitimacy, sufferers look to another pain that is sufficiently recognized by society as a point of reference for their own. By way of example, myalgic encephalomyelitis is compared with multiple sclerosis, obesity with diabetes, bereavement with recovering from a heart attack, and all pains are compared with cancer. See Box 24.

**Box 24**

If a cancer patient overcomes the acute phase, they say, “now I’m clear. I don’t have cancer anymore.” Right? But the obese are always obese [5B81]

Like cancer, where you see the effects immediately. People don’t tend to judge those kind of things [M630.01]

Strategies to gain legitimacy are also deployed collectively to overcome stigmatization, such as in the case of some mental illnesses, but mainly to obtain public funds inaccessible to those affected by types of pain relating to contested illnesses or illnesses that lack institutionalization in the healthcare system. Mutual support groups are organized, for example, to deal with the pain of the loss of children, for children in situations of adoption, or the victims of bullying. One of the aims of patient associations is to incentivize research on the pain affecting them, but the main aim is to gain legitimacy.

### How the legitimacy of pain works in practice

As pain is a relational concept, its legitimacy is a process that is negotiated in terms of formal recognition and resource allocation within the welfare state. At times this negotiation is individual, but generally speaking pain does not obtain either normative or moral legitimacy until this is negotiated in an organized and collective manner, via associations of sufferers and patients or as a consequence of concrete social events, (for example, the recognition of certain syndromes in soldiers returning from the various wars of the twentieth century [22].

Social fields and environments always necessitate the fulfillment of some further requirement in addition to normative recognition, whether because custom requires it or because dominant social representations act as a model and it is necessary to resemble them.

Legitimacy becomes a complex negotiation process in which some stand to win more than others, but no win is absolute. Inputs vary depending on the pain and the field in which legitimacy is being negotiated, although in practice the results are similar, since any legitimacy obtained is partial or conditional and comes with an expiry date. In other words, no pain is ever fully legitimated, and legitimacy does not last forever.

All legitimacy expires, and time is not the only determining factor [4A40]. See Box 25.

**Box 25**

But at this point—after so long, after six years—there are still people who don’t understand you [4A40]

One of the reasons it expires is the sense of exasperation resulting from failed attempts to help the sufferer. Legitimacy deteriorates as a result of the belief that the sufferer is a willing participant in their situation [CSSGBV]. See Box 26.

**Box 26**

[A victim of gender-based violence states] my mum is very difficult. Seeing as I had made two formal complaints about this person [abuser], and then withdrew them along with the restraining order and everything and got back together with him, […] my mum didn’t want to take me in […] I was living in a rented garage and my mum said that as long as I was with him she didn’t want to have anything to do with me [CSSGBV]

In other instances, pain is viewed as attention-seeking behavior aimed at manipulating others, and thus any legitimacy it may have had disappears [MB245]. See Box 27.

**Box 27**

Tell me you don’t want to hear about it, that you’re tired—it’s fine. I don’t know, anything would be better than hearing, “stop trying to be the center of attention.” That’s really hurtful [MB245]

Even the legitimacy surrounding the pain of losing a daughter expires when the mourning period does not unfold in line with society’s expectations: when it goes on for a long time or passes only to return, manifests during leisure activities, or interferes with colleagues’ work [EIDD]. See Box 28.

**Box 28**

And people just don’t understand that you. . . [People say] “But are you both still feeling like that? [. . .] After two years, [the mourning period] should be over, right?” […] My wife feels embarrassed to go to the mall, for example, because she might bump into […] pupils or colleagues. [The colleagues will be thinking] “Why don’t you come back to work? Stop expecting to get paid. Stop being a problem” [EIDD]

In general, pain is deemed to have passed its expiry date once—subsequent to a grace period—it begins to have a negative impact on social productivity [EIRB]. See Box 29.

**Box 29**

I mean, you don’t just lose your husband; you lose other people along the way, which is additionally painful […] I think you become. . . you’re no good to them anymore. If you go down that road for a long time, you’re no use to them [EIRB]

Even for the most understood and legitimated types of pain, there is always the hint of a suspicion that it may be faked. Indeed, the fact that legitimacy is not open-ended makes suspicions permissible by society.

In the context of the partial legitimacy ultimately conferred upon pain, sufferers believe that some kinds of pain are more legitimated than others, although all believe that their own pain is not legitimated.

According to the narratives, pain with visible effects benefits from greater legitimacy since it is demonstrable. Suffering that can be objectified, even if it is not visible, also enjoys greater legitimacy than that which cannot. Pain related to cancer, death, or the possibility of dying or being a person with a disability is also highly legitimated.

People with pain whose impact cannot be seen, initially continue with their daily activities, which allows them to keep legitimacy as sufferers. As time goes by people start to think that the pain cannot be that bad if the sufferer can go on doing what they did before. The initial legitimating effort turns into suspicion from others. This situation responds to a significant paradox in order to gain legitimacy: pain is perceived as legitimate if it does not interfere with daily activities, but by not interfering, it is assumed that: either the pain does not exist or it is not that bad. Other narratives focus neither on the pain nor its origins when assessing legitimacy, but rather on the type of relationship established with the sufferer. The closer or more affectionate the relationship, the more legitimate the pain is perceived to be. Even when two people suffer the same pain, the suffering of the person we feel more affection for will be considered more legitimate.

Through their narratives about pain, individuals reveal the existence of relations of domination that expel them from the social spaces they occupy and in which they have ceased to be useful. This situation will be called the pathology of “helplessness” [63].

## Discussion

This study has explored the relationship between pain and legitimacy in different areas of the social structure through the perceptions of those with chronic pain of various origins.

The narratives show that pain is a social phenomenon that is constantly subject to a process of social evaluation that places it in the gray area of the legitimacy-illegitimacy continuum [39], that is, in a state of partial legitimacy [64] p. 345 cited in [23].

The IASP’s definition of pain and the rise of the biopsychosocial model [12] facilitate an interpretation of pain as a phenomenon with a social and historical dimension [2–22] that must be treated, as well as emphasizing the importance of social context for pain [17] cited in [23].

We first looked at the role of expert systems [58] in their capacity as distributive sources of legitimacy. Sufferers encountered ineffective dynamics that failed to facilitate normative legitimacy, especially where research into how to treat a particular pain is lacking. According to the literature, difficulties exist in part also because the systems answer to a model of bureaucratic rationality [65] that clashes with sufferers’ practical rationality [59]. This is why, despite the apparent support given by these systems, the relationship between sufferer-user and expert hovers somewhere between trust and suspicion [66]. In this social space, pain resulting from contested illnesses [67, 68] from an emotional impact, or from complex social situations lacks known legitimacy given an absence of institutionalization and knowledge, and the deviation from social expectations [39].

At the same time, decades-old social discourses exist which analyze effectiveness, efficiency, and productivity in relation to illness [69] and urge sufferers not to get in the way of these goals. This article shows that workers are keen to underline that they have no intention of letting their company down; indeed, when they can, they compensate for the decrease in their productivity. The interviewees questioned their own legitimacy in relation to pain [23] and so developed strategies to gain this to some degree.

On the other hand, changes to the model of work mean that vulnerability and poverty have become a real threat for some workers [70], especially those with poorer health and worse working conditions. In this sense, it is almost impossible to obtain legitimacy if the pain necessitates a reduced workload or even a temporary leave of absence. Sufferers with worse working conditions say they lack any real opportunity to take care of their health or treat their pain. Despite also being doubted, those with better working conditions have more opportunities to treat the pain.

Pain in relation to work shows that fulfilling [32] “sick role” is not enough to gain legitimacy in cases of chronic pain, given that the requirement of temporariness is replaced by that of permanent incapacity, altering the social contract that had existed up to that point [71] cited in [23].

Analyzing fibromyalgia in relation to the American discursive context around hard work, Paxman [72] observes how sufferers struggle in order not to be considered lazy. In the field of work, the narratives show that inhibition and disbelieve of people in pain are widespread due to its impact on productivity, with suspicion of pretense a key factor.

The narratives show that sufferers know their pain will be doubted via different strategies [73] in order to maintain the current economic system. But their motivation and need to comply and not let the company down is also clear to see, which [74] understands as a mechanism for maintaining productivity. In this sense, workers whose pain is legitimated in their respective companies express a sense of privilege relative to other workers in a similar situation.

In all these processes, sufferers have their testimony and credibility doubted in various ways, and they may even experience rejection as a result. In particular, unrecognized pain is subject to hermeneutic injustice [39].

Individuals develop strategies to legitimate the pain they experience. They strive to express the “reality of the illness,” relating their experiences and emotions in order that society at large might share them and make sense of them [75]. Furthermore, they are convinced that recognition of their pain will have benefits for society as a whole.

Hypothetically, pain lacks legitimacy where it results from illnesses that cannot be detected by standardized diagnostic medical tests [25]. However, this study shows that even where pain is experienced in the context of a known and institutionalized illness, or as a result of a socially respected and undisputed situation, its legitimacy is not absolute, and moreover must be obtained by the sufferer. In other words, all illnesses, no matter how well known, are faced with varying degrees of illegitimacy and are thus situated with in the grey area of legitimacy [39].

This begs the question: Which is the object of legitimacy, the pain or the sufferer? Pain and sufferer cannot be separated, however, doing so has the analytical purpose of facilitating the observation of processes that could not be observed otherwise. Given that legitimacy can only be analyzed in concrete situations [76] and in the context of social relations, analysis of the specific situations of these sufferers and their pain shows that both face obstacles to legitimation [39] but via different processes. Whereas the individual is assessed in their interpersonal relationships via testimonial injustice and social control, pain is questioned in terms of its legitimacy as a social phenomenon in its formal aspects via social and institutional processes.

Social and scientific debates are currently underway concerning the medicalization of biological processes [77], the stigmatization of certain conditions [78], and the nature of behavioral disorders [79]. Sufferers see medicalization as guaranteeing the legitimacy of their situation, since this enables them to access quality care and resources from the welfare state.

They are concerned by some aspects of legitimacy. First, with that of recognition [14–80], in the light of stablished norms, values and beliefs in order to be treated as other sufferers. Second, they are concerned by regulations in “legal sphere” about redistribution [81] of resources from the welfare state.

In terms of the need to gain legitimacy, this is common among all sufferers, regardless of their type of pain. They all enact pre-analyzed strategies at an individual level, as well as collective strategies such as the forming of patient associations, healthcare lobbying, or the funding of specific studies. In this sense, both strategies can be understood, since they are in line with the social order in which these individuals live [82] that is, a strongly individualized system in which the state is losing relative importance and is showing limitations in terms of covering old and new social risks [60]. In this way, individual and collective strategies connect with the system of social values, norms, and expectations [39].

The legitimation of pain is a process involving interactions and judgments of individuals, social processes, and supraindividual processes [38] cited in [35], and is rarely produced.

Conversely, there are forces that offer resistance to the legitimization of pain. First, the flexibilization of the labor market has diminished labor rights and social risk coverage for workers. This same trend also stems from the retreat of the welfare state, making individuals responsible for meeting their own needs and, therefore, managing their health and pain.

On the other hand, the strength of social roles is such that when pain interferes with their fulfillment, the social reaction is channeled toward critical judgments that prevent the legitimation of pain and its agent—the individual—and toward suspicion.

## Conclusions

This study provides a comprehensive explanation of the reasons why pain is subjected to processes that prevent it from gaining complete legitimacy. The narratives show that all types of pain, regardless of their origin and manifestation, are delegitimated in some of the contexts in which they manifest.

They also demonstrate that in order to obtain legitimacy, sufferers develop a set of strategies and comply with a series of norms that can be contradictory. Legitimacy is constructed separately in each area of life, the particular demands of which can contradict each other. Moreover, legitimacy is always subject to an expiry date, whereby whatever legitimacy has been obtained will be lost.

Ultimately, our research shows pain to be a highly questioned social phenomenon owing to its severe impact on productivity and the maintenance of daily activity.

The experience of chronic pain in its various manifestations, however well known, shows it to be incompatible with the demands of contemporary complex societies, for which productivity is a legitimating principle of the functioning of the social order.

Social pacts in relation to pain have changed since Parsons’ [32] notion of the sick role. Today, pain is a disruptor of the system which cannot be completely legitimated because it hinders compliance with what are functional processes: roles that affect the economic system and the sexual division of labor (production and reproduction), and processes that maintain culture and consumption (traditions, leisure, and free time).

Through their experiences, the interviewees bring to light the existence of a series of relations of domination that expel them from the social spaces they occupy, in which they cease to be useful. Giddens [64] would call this situation a pathology of “helplessness”.

Granting pain and those who experience it a state of complete legitimacy would require the establishment of a new social contract.

## Supporting information

S1 File COREQ. Consolidated criteria for reporting qualitative research.
(DOCX)

S2 File Coding tree. (DOCX)

S3 File Semi-structured interview script. (DOCX)
