# Supplementary material for: The legitimacy of pain according to sufferers
Source: PLoS One. 2023 Nov 15;18(11):e0291112. doi: 10.1371/journal.pone.0291112 (PMC10651017; doi:10.1371/journal.pone.0291112)
Supplement: S1 File — Consolidated criteria for reporting qualitative research. (DOCX) [file pone.0291112.s001.docx]

COREQ (Consolidated Criteria for Reporting Qualitative Studies)

| N_o Item_ | Guide question/description | Reported |
| --- | --- | --- |
| Domain 1: Research team and reflexivity | | |
| Personal Characteristics |  |  |
| 1. Interviewer/facilitator | Which autors conducted the interview? | The interviews were conducted by twoo interviewers from the research team with Experience and training in qualitative research |
| 2. Credentials | What were the researcher’s credentials? | The researcher’s credential are Both PdD |
| 3. Occupation | What was their occupation at the time of the study? |  |
| 4. Gender | Was the researcher male or female? | The interviews Were conducted by one woman and one man both researchers |
| 5. Experience and training relationships with participants | What experience or training did the researcher have? | The two researchers have extensive training and experiences in research using qualitative research |
| 6. Relationships established | Was a relationship established prior to study commencement? | No |
| 7. participant knowledge of the interviewers | What did the participants know about the researcher | Participants received verbal and written information about the research and its objetives, the research group and the affiliation of each one, and about the funding institution. |
| 8. Interviewers chatacteristics | What characteristic were Reported about the Interviewer/facilitator? |  |
| Domain 2: study design  Theoretical framework | | |
| 9. Metodological orientation and theory | What metodological orientation was stated to underpin the study? | Content analysis |
| Participant selection | How were participant selection? | The participants were selected following the Sampling criteria. The procedure was to search in organizations specialized in a particular pain. A specialized Company was hired to search for the least accesible profiles |
| 10. Sampling |  | Purposive. MVS.  The interviewees were selected to fullfil a profile: experiencing a specific pain related to its origin. |
| 11. Method of approach | How were participants approached? | A review of the associations of those affected by pain was carried out to select the most appropriate for the object of study. Once selected, they were contacted to request contact with a person with the profile of our interest. Obtaining the contact of the interviewee, each one of them was contacted through an email in which the characteristics of the research, its objectives, those of the research team and why their participation was necessary were explained. Once they agreed to participate, they were contacted by telephone to determine the conditions of the interview: date, place, time, etc. and provide them with the information in writing, as well as request their informed consent. |
| 12. Sample size | How many participants were in the study? | 19 interviewed: five of then in pain from physical origin; four, in pain from psychological origin; four in pain from emotional origin; four in pain from complex social situations. . |
| 13. Non-participation setting | How many people refused to participate or dropped out? Reasons? | There was no refused. |
| 14. Setting of data collection | Where was the data collected? | The interviews were carried out in three ways: face-to-face, online and by telephone (Because of the confinement produced during the pandemic). |
| 15. Presence of non-participants | Was anyone else present besides the participants and researchers? | No |
| 16. Description of sample | What are the important Characteristics of the sample | The heterogeneity of the sample seeking to include a wide variety of pain:  1. Include all sources of pain  2. In each origin include different pains  3. Include men and women  4. Include a variety of ages  5. Geographic representation. The interviews have been carried out in seven autonomous communities, Andalusia being the Community in which the most interviews have been carried out, as it is the Community with the largest population. Within Andalusia, they have also been distributed in the different provinces. |
| Data collection |  |  |
| 17. Interview guide | Were questions, prompts, guides provided by the authors? Was is pilot tested? | The interview scripts were designed by the research team in team meetings. They were piloted and tested at the beginning of the field work. |
| 18. Repeat interviews? | Were repeat interviews? | No |
| 19. Audio/visual recording | Did the research use audio or visual recording to collect the data? | All interviews were audio-recorded at the time they were conducted. |
| 20. Field notes | Were field notes made during and/or after the interview or focus group? | Field notes were made after the interviews. Recogen el contexto de la entrevista y algunas ideas para el análisis. |
| 21. Duration | What was the duration of the interviews or focus group? | The average duration of the interviews was 80’60” |
| 22. Data saturation | Was data saturation discussed? | The research team considered that the saturation of the field occurred when the las interviews did not provide new information to that already provide new information to that already provided by the previous ones. |
| 23. Transcripts returned | Were transcripts returned to participants for comment an/or correction? | No |
| Domain 3. Analysis and findings |  |  |
| Data analysis |  |  |
| 24. Number of data coders | How many data coders coded the data? | One of the researchers carried out the preliminary coding that was supervised, discussed and agreed with two other researchers from the research team. |
| 25. Description of the coding tree | Did authors provide a description of the coding tree? | Yes, we provide the description of the coding tree |
| 26. Derivation of themes | Were themes identified in advance or derived from data? | The categories and codes used are derived: some from the research objetives, therefore they were available from the beginning of the analysis process. Others ones were derived from textual analysis and were incorporated into the analysis and the coding tree. |
| 27. Sofware | What software, if applicable, was used to manage the data? |  |
| 28. Participant Checking Reporting | Did participants provide feed back on the findings? | Given the subject of the investigation, the interviews were very hard for the interviewees, so we decided not to propose new meetings to receive feed back. |
| 29. Quotations presented | Were participant quotations presented to illustrate the themes/findings? Was each quotation identified? | Identified quotations are included in results section and have been identified guaranteeing the anonymity of the interviewee |
| 30.Data and findings consistent | Was there consistency between the data presented and the findings? | Yes |
| 31. Clarity of major themes | Were major themes clearly presented in the findings? | Yes |
| 32. Clarity of minor themes | Is there a description of diverse cases or discussion of minor themes? | Yes |

Source: Compiled by the author
