# Supplementary material for: The legitimacy of pain according to sufferers
Source: PLoS One. 2023 Nov 15;18(11):e0291112. doi: 10.1371/journal.pone.0291112 (PMC10651017; doi:10.1371/journal.pone.0291112)
Supplement: S2 File — (DOCX) [file pone.0291112.s002.docx]

Coding Tree.

| CATEGORY | CODE | DESCRIPTION |
| --- | --- | --- |
| PROCESSES OF SOCIAL ILLEGITIMISATION  OF PAIN | Los distribuidores de legitimidad.  Institucional-formal. | Expert systems acting as mediators between the sufferers and society: Health system, INNS, Education system, Judicial system. |
|  | The working field | Difficulties encountered in the workplace in managing pain.  The suffererʹs productivity  Relationship of oneʹs own pain to co-workersʹ productivity.  Relationship of oneʹs own pain to work reorganisation. |
|  | Epistemic injustices | Injustices that occur in the testimony of people with pain in their interpersonal relationships that question, doubt, suspect or deny testimonies about pain. |
| PERCEPTION OF THE LEGITIMACY OF PAIN ACCORDING TO THE SUFFERERS | Marks of illegitimacy | Each pain has a specific bran associated with it. |
|  | Painʹs position on the “illegitimacy – legitimacy” axis | Perception of the pains that enjoy more social legitimacy and those that enjoy less and their justification. |
|  | States of legitimacy in "the gray area" (Siraz, 2022 ) | Situations in which the legitimacy and illegitimacy of pain are not absolute. There are elements that place them in an intermediate space. |
| SUFFERERSʹS ESTRATEGIES FOR LEGITIMISING PAIN | Maintenance of social structure Diagnosis and behavior | Not to alter the social order by playing the social roles and respecting the social norms that correspond (in one part only) to the “sick role” (Parsons) |
|  | Relacional | Discursive strategies and actions to justify the situation derived from the pain. |
|  | Keeping oneʹs legitimacy | Use of silence. Not to stand out so as not to attact attention, thus avoiding the unleashing of delegitimising forces. |
|  |  | Principle of non-interference in each otherʹs contexts. |
|  |  | Performance of social roles |
|  |  | Maintenance, increase or compensation of productivity in social spheres in which the sufferers participate |
| BASIC RULES OF LEGITIMACY | The nature of legitimacy | How is the legitimacy of pain established.  Is it a quality of pain or is it established at the level of social relations? |
|  | Length of legitimacy | Changes produced in the legitimacy of pain over time or due to the behaviour of the sufferers |
|  | What is delegitimised, the sufferers or the pain? | Where does legitimacy lie, in the pain itself or in the person in pain? |
| Source: Compiled by the author | | |
