# Supplementary material for: The legitimacy of pain according to sufferers
Source: PLoS One. 2023 Nov 15;18(11):e0291112. doi: 10.1371/journal.pone.0291112 (PMC10651017; doi:10.1371/journal.pone.0291112)
Supplement: S3 File — (DOCX) [file pone.0291112.s003.docx]

Semi-estructured interview script

| Main dimensions | Questions |
| --- | --- |
| First contact. Introduction of the inverviewer. | Introduction of the inverviewer and the interviewee  Explain the objetives of the interview to the interviewee  Ask for informed consent from the interviewee |
| 1. Inervieweeʹs current situación | 1.1 How are you currently feeling? |
| 1. Onset and course of pain | 2.1 When did the symptoms start?  2.2 How did the symptoms start?  2.3 Can you identify the triggering element of the situation?  2.4 ¿How did you feel in that situation? |
| 1. Description and behavior of pain | 3.1 Could you describe What that pain is like?  3.2 How has the pain been progressing during this time?  3.3 How often do you usually have relapses, crises or other pain-related situation?  3.4 How long do crises or relapses usually last?  3.5 Has pain changed over time? |
| 1. Coping with pain | 4.1 How do you cope with pain, how you deal with it on a day-to-day basis? |
| 1. The diagnosis | 5.1 ¿Do you have a medical diagnosis about you pain or the situation you relate to?  5.2 Has de diagnosis had any influence on the pain? |
| 1. Impact of pain on work, family and social life | 6.1 Do you have a job?, How does pain affect your work?  6.2 What impact has the pain had on your work, family and social life?  6.3 How has the pain affected your social life, relationship with friends, leisure activities, free time?  6.4 Have treatments or other pain-related needs caused you any financial problems?  6.5 What impact has pain had on your overall quality of life? |
| 1. Social attitudes to pain | 7.1. ¿How was your pain dealt with in the health care system?; Was your situation ever questioned?  7.2. How has your work environment reacted to your pain?  7.3 ¿How has your family reacted to your pain?  7.5 ¿Do you think that your pain and the circumstances that cause it are understood by society as a whole?  7.6 Have you ever felt that they doubted your circumstances?, Do you think they thought you were exaggerating your circumstances?  7.7. Do you think that people eventually grow tired of those in pain?  7.8 Would it be posible that your circumstances would be less believed or understood if they were a nuisance to someone else, be ir a family member, friend or co-worker?  7.9 Have you ever felt stigmatised because of you pain or health?  7.10 Do you know any stereotypes or myths about your pain or illness? |
| 1. The most legitimised and the least legitimised pains | 8.1 What would you say is the most respected pain in society?  8.2. And in you opinión, which one is the least respected?  8.3 Do you think that some people are believed more than others when they are affected by pain or illness?  8.4. Do you feel understood when it comes to your pain?  8.5. ¿What do you think is the hardest pain you can have? |

Source: Compiled by the author
